# Comparison of Two Levels of Pressure Support Ventilation on Success of Extubation in Preterm Neonates: A Randomized Clinical Trial

**DOI:** 10.5539/gjhs.v8n2p240

**Published:** 2015-06-25

**Authors:** Roya Farhadi, Hamid Reza Lotfi, Abbas Alipour, Maryam Nakhshab, Vajiheh Ghaffari, Seyyed Abbas Hashemi

**Affiliations:** 1 Pediatrics Department, School of Medicine, Mazandaran University of Medical Sciences, Sari, Iran; 2 Community Health Department, School of Medicine, Mazandaran University of Medical Sciences, Sari, Iran

**Keywords:** pressure support ventilation, neonate, mechanical ventilation, weaning

## Abstract

**Background:**

Pressure Support Ventilation (PSV) is one of the modes of mechanical ventilation that can be used alone as a weaning strategy in neonates. However, studies on the appropriate pressure level for this mode in neonates are limited.

**Objectives:**

Because the use of adequate pressure support in this mode, keeping the appropriate neonate’s tidal volume, and preventing the respiratory complications, this study was aimed to compare extubation failure in the two levels of pressure support ventilation of 10 and 14 cmH2O when removing the neonates from the ventilator.

**Materials & Methods:**

In this randomized clinical trial 50 premature infants of 27-37 weeks with respiratory distress syndrome (RDS) were under mechanical ventilation for at least 48 hours, were randomly assigned to two groups. One group was extubated in PSV mode with pressure of 14 cmH_2_O and the other with 10 cmH_2_O. Extubation failure rate and complications such as pneumothorax, death and respiratory parameters were compared in the two groups.

**Results:**

Twenty five neonates in each group were assessed. Weaning time, extubation failure rate, and mean airway pressure was lesser in PSV of 10 cmH20 group than Level of 14 cmH2O and those differences were statistically significant (P<0.05). Difference between work of breathing, ventilation time, pneumothorax and mortality rate between two groups were not statistically significant (P>0.05).

**Conclusion:**

The results of our study show that extubation of the neonates using 10 CmH_2_O in PSV mode increases the success rate of extubation. Although when Volume- assured PSV can be used, it is more logical to use it for guaranteeing tidal volume, but using the appropriate level of pressure support when the PSV mode is used alone is inevitable and further studies are necessary to demonstrate the level of pressure in this mode.

## 1. Introduction

Advances in neonatal intensive care and mechanical ventilation of newborns have increased infants’ survival during recent years and was subject of many recent investigations (Donn et al., 2003; Wu et al., 2015; Armanian et al., 2014; Salvo et al., 2015; Chu et al., 2015; Akinloye et al., 2014; Schmölzer et al., 2014). New ventilation methods and techniques that have newly become available have made the mechanical ventilation in neonates a masterpiece in the twenty-first century (Keszler, 2006). Development of microprocessor technology and pressure and flow transducers has made significant progress in the design of ventilators and new modes have been invented (Smith et al, 2010; Greenough et al, 2005).

PSV (pressure support ventilation) is one of the mechanical ventilation modes used in infants who have spontaneous breathing, and its use have been available since 1990, and is designed to overcome work of breathing by strengthening inspiratory pressure of respiration (Sarkar et al., 2007). This mode is often used in combination with SIMV (Synchronized Intermittent Mandatory Ventilation) mode as a Weaning strategy, but can be used as a stand-alone mode in patients who have reliable breathing and is also used for prevention and treatment of broncho-pulmunary dysplasia (BPD) due to its low pressure (Sarkar et al., 2007; Masahiko et al., 1998). In this mode, the level of pressure support would be determined by the clinician and the respiratory count supported can be complete, partial, or very low. Flow amounts delivered vary with patient’s respiratory effort (Donn, et al, 2003). Patient’s respiration is sensed by a pressure or flow (most commonly) sensor while a maximum inspiratory time is also set (Hummler et al., 2009). If the infant gets apnea a mandatory Back-up rate would be settled to enable ventilation continuity (Claure, 2007). Thus, PSV unlike other modes, has only minimal complexity, because only the settings settled have the pressure support level (Serra et al., 2005).

Pressure support ventilation can be usually kept in 30-50% difference of Peak Inspiratory Pressure (PIP) and Positive End Expiratory Pressure (PEEP) (delta pressure). If the pressure is set exactly at the PIP pressure, it would be same as Assist Control (AC) mode, with this difference that in PSV mode, the inspiratory time would be determined by the patient (Sarkar et al., 2007; Ramanathan, 2005). Though the clinician can limit the inspiratory time to a maximum that inspiration can be terminated with patient’s respiration flow or settled inspiratory time (Max Ti), whichever occurs earlier (Sarkar et al., 2007).

Despite various studies regarding using PSV mode in adults, few studies are available for using this mode in neonates. Some studies performed with small sample sizes have suggested its safety as a stand-alone mode even in very low birth weight premature infants; however, researchers recommended further clinical trials for more evidence-based using of this mode. (Sarkar et al., 2007) The pressure needed in this mode is lower than conventional PIP and some sources have mentioned that the pressure must be regulated between 10-15 cmH_2_O to overcome work of breathing of small endotracheal tube(ETT) and ventilator circuits and it is not necessary to decrease pressure support levels below 10 cmH2O (Smith et al., 2010; Adamz et al., 2013). Regarding the fact that studies assessing the proper pressure levels in this mode in neonates are very few (Adamz et al., 2013) and most of this pressures are investigated in mixed modes, and the use of adequate protection in this mode, keeps the appropriate neonate’s tidal volume, and prevents the respiratory complications, this study was aimed to compare extubation failure in the two levels of pressure support ventilation of 10 and 14 cmH_2_O when removing the neonates from the ventilator in this mode.

## 2. Materials and Methods

### 2.1 Ethics

This study is a randomized clinical trial (RCT) was conducted in the level III neonatal intensive care unit of Bu-Ali Sina hospital in Sari (north of Iran) between October 2013 to December 2014, with approval of the Ethics Committee for Research of Mazandaran University of Medical Sciences and has been recorded in the Iranian registry of clinical trials by the code IRCT201309302801N3.

### 2.2 Inclusion and Exclusion Criteria

the studied infants included premature infants between 27-37 weeks with Respiratory Distress Syndrome (RDS) during the first week of their lives who were under the ventilator therapy and were mechanically ventilated for at least 48 hours.

Infants with pneumothorax, congenital anomalies, simultaneous meconium aspiration pneumonia, congenital heart disease, symptomatic PDA, asphyxia, interventricular hemorrhage grade III and IV and infants using sedative drugs or had leakage more than 20% from ETT were excluded.

### 2.3 Study Protocol

Infants entered into the study received flow triggered ventilator therapy by SLE4000 infant ventilator (SLE Ltd. UK) in synchronized intermittent mandatory ventilation mode (SIMV), while routine care of NICU was performed in a standard protocol. Once the ventilator respiratory rate reaches 20/min, PIP reaches 16cmH2O, PEEP of 4cmH2O, and FIO2<30% and blood gases and clinical condition of neonates were acceptable (12) the ventilator mode changed to PSV. After explaining the protocol to the infants’ parents, written informed consent was obtained and eligible patients were randomly assigned and recruited into the study as parallel two groups.

Infants were randomly assigned into two groups. One group was extubated in PSV with pressure support of 14 cmH_2_O and the other with 10 cmH_2_O with maximum inspiration time of 0.5 seconds and backup rate of 15 per minute with trigger sensitivity of 100%. After at least 24 hours of stabilization in this pressure level, infants were extubated in similar care and technical conditions, in case of acceptable blood gases (Ph>7.25 and PCo_2_<60 mmHg) and stable clinical condition of neonates. Hemoglobin in all infants was kept at the level of 13 gr/dl. Infants with recurrent apnea, respiratory acidosis (Ph<7.2 and PCO_2_>60mmHg) or respiratory rate of higher than 80per minute during the study were excluded from the study. SLE4000 ventilator was equipped with a screen for displaying mean airway pressure (MAP) and tidal volume and work of breathing.

Four hours before the extubation, the Feeding were withheld, and if they were under mechanical ventilator for more than 7 days, dexamethasone was prescribed 24 hours before extubation. For infants less than 34 weeks, Aminophylline was prescribed, when ventilator rate was equal to or less than 30 minutes and all the prescribed analgesics, sedative or muscle relaxant were interrupted at least 24 hours before extubation and simultaneous to starting PSV mode.

Randomization was performed by concurrent active form and put in sealed envelopes (pressure support) which were delivered to resident of the team by the project nurse during PSV beginning mode. After extubation of infants, they were positioned in the prone position and remained NPO for 24 hours. Routine nursing care such as suction was performed equally for both groups according to our ward routine nurses’ protocol. After extubation, the infants were monitored for vital signs, oxygen saturation percentage for at least 72 hours and until full stability.

For each infant, birth weight, age, gestational age (which were extracted from ultrasonographic data documented in mother’s files, or if not available were determined by a neonatologist according to Balard scoring system). The age of beginning ventilator therapy, days under ventilator therapy, Weaning duration (time from PSV starting to extubation), use of surfactant, and administration of steroids before birth were recorded. Need for re-intubation (due to repeated episodes of apnea, hypoxia, and hypercapnia) within 48 hours of extubation was recorded as extubation failure as a primary outcome of our study (Donn, et al, 2003).

Other outcomes measured included overall time of ventilator therapy, pneumothorax or death within 72 hours after extubation, duration of ventilation during PSV mode in hours (Weaning duration), median of tidal volume during PSV mode and work of breathing (based on registration of nurse every two hours). Median of total respiratory rate (based on registration of nurse every two hours) was also documented.

### 2.4 Statistical Analysis

We used the Shapiro-Wilk test to test whether data were normally distributed. Descriptive baseline characteristics for two groups (Level of 10 cmH20 for pressure support group and Level of 14 cmH20 for pressure support group) comparisons were tabulated as median (inter-quartile range) or as percentages. Comparing between two groups for categorical data were statistically analyzed using chi- square or Fisher-exact test and for continuous data were statistically analyzed using mann-whitney U test. A p value of 0.05 or less was considered statistically significant. Data were analyzed using IBM SPSS statistics version 16 and Stata version 10.

## 3. Results

**Figure 1:**
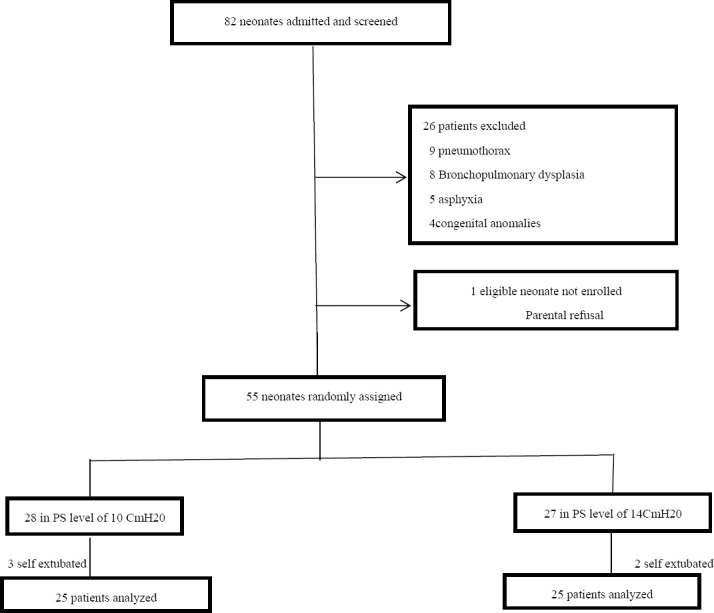
Flow of randomization and enrollment process of study

In this RCT which was done from October 2013 to December 2014, a total of 82neonates with respiratory distress syndrome (RDS) were referred to our hospital. Of these, 26 patients did not meet the inclusion criteria and 1 patient declined to participate in the study. From 55 patients who allocated in the two groups (10 and 14 cmH2o of supporting pressure level), 3 and 2 patients losses to follow-up during study period in 10 cmH2o and 14 cmH2o group, respectively. In total, 50 patients completed the present study and data from all these patients were analyzed (Figure 1).

Basic demographic and clinical characteristics of neonates and their gestational state in two groups are presented in Table 1. Results shows that difference of variables were not statistically significant (p>0.05).

**Table 1:** Basic demographic and clinical characteristics of neonates {Data are expressed as the median (inter-quartile range) or as number (percentages)}

Variables		Group of study		P value

		Level of **10 cmH20** for pressure support	Level of **14 cmH20** for pressure support	
Age at extubation time (days)		9 (7-15)	8 (6-15.5)	0.45
Sex (M/F)		11/14	12/13	0.88
Weight (gr)		1750 (1200-2125)	1800 (1285-2025)	0.79
Gestational age (weeks)		31 (29-33.5)	32 (29-34)	0.74
Kind of delivery	C/S	24 (96 %)	23 (92 %)	>0.99
	NVD	1 (4 %)	2 (8 %)
antenatal steroids		14 (56 %)	12 (48 %)	0.53
surfactant		19 (76 %)	17 (68 %)	0.57

Table 2 shows the median (inter-quartile range) or number (percentages) values of the outcome parameters of each group. As shown in Table 2, weaning time, extubation failure rate, and MAP was lesser in Level of 10 cmH20 for pressure support group than Level of 14 cmH20 for pressure support group and those differences were statistically significant (P<0.05). We showed that Although other outcome variables were better in Level of 10 cmH20 for pressure support group than Level of 14 cmH20 for pressure support group but were not statistically significant differences between two groups (P>0.05).

**Table 2:** Outcome variables in two levels of pressure support ventilation Data are expressed as the median (inter-quartile range) or as number (percentages)

Variables	Group of study		P value

	Level of **10 cmH20** for pressure support	Level of **14 cmH20** for pressure support	
Duration of ventilation(days)	7 (5-8.5)	7 (3.5-9)	0.73
Weaning time (hours)	24 (21-24)	48 (27-51)	**<0.0001**
**Work of breathing**(joules)	178 (172-321)	240 (163-490.5)	0.18
Tidal Volume(mL)	8.7 (5.5-18.1)	15.1 (4.55-17.4)	0.54
Extubation failure	1 (4%)	6 (24%)	**0.049**
Pneumothorax	2 (8 %)	6 (24 %)	0.12
Death	0 (0 %)	0 (0 %)	>0.99
Total rate/min	42 (37.5-47)	42 (37-53.5)	0.71
*MAP(cmH2O)	4 (4-5)	5 (4-5)	**0.025**
Minute ventilation (mL)	0.31 (0.14-0.51)	0.39 (0.17-0.44)	0.96
respiratory rate/tidal volume	2.8 (2.3-8.8)	3.4 (2.45-6.7)	0.71

* MAP: Mean Airway Pressure.

## 4. Discussion

The results of our study showed that gradual reduction of the peak inspiratory pressure and converting it to pressure support of 10 cmH_2_O significantly increases extubation success rate compared to the pressure support of 14 cmH_2_O and the neonate can be extubated from PSV mode in shorter period.

Several studies have investigated the use of this mode combined with the SIMV mode. Reyes et al (2006) have compared SIMV and SIMV + PSV in a clinical trial and its impact on weaning from mechanical ventilation and concluded that the mixed mode requires fewer setting of ventilator and infants were extubated earlier from SIMV alone. But the general duration of ventilation therapy did not differ between the groups like our study. This study was performed on preterm infants less than 1000 grams and the pressure support was set at 50% of difference of PIP and PEEP. Though the main goal of this study was not weaning, and it aimed to reduce the exposure to higher rates of SIMV mode.

Osorio et al. (2005) assessed the SIMV mode alone with two levels of pressure support with SIMV in 15 preterm infants. The two support levels were set at level 3 and 6 cmH_2_O and reduction in respiratory rate in SIMV mode were compared between the two pressure levels and the conclusions was that support of 6 cm H_2_O resulted in a 50% reduction in SIMV rate and tidal volume in mixed mode was more than the pure mode, but Pco_2_ and FIO_2_ were not significantly different in three methods. The authors believed that although PSV can be used alone, it can cause hypoventilation in periods that the respiratory effort is week and recommended further studies to be done in this regard (Osorio et al., 2005).

In another study Gupta et al (2009) compared the effect of two level of pressure support on the tidal volume and minute ventilation in phase of separating from the ventilator on 10 infants less than 32 weeks and in this study, 3 modes of SIMV alone, SIMV + PS max and SIMV + PS min were compared and it was found that using Psmax (generator of minute volume of 8 cc/kg) increased the total minute ventilation in comparison to the synchronized mode alone and this difference also existed in comparison of PS min with synchronized mode. The authors believed that using PSV appropriate to pressure level can stabilize the patient’s breathing. In any case, this study was not done in PSV mode alone and combination mode was compared. Contradictory to this study, our study showed that in PSV mode alone no significant change has been established in minute ventilation

However, although there are similarities between our results with aforementioned studies on the usefulness of utilizing PSV mode, it is clear that the above mentioned studies did not investigate PSV mode alone and their sample size was also relatively small. The main point is that although the PSV as a weaning technique is mentioned alone, these studies are very limited in neonates and another point is that the optimal support pressure is also not yet clear as in adults (Adamz et al., 2013).

Coddington et al. (2000) retrospectively examined levels of pressure support in 100 patients before extubation to determine the appropriate pressure on PSV mode for separating tracheal tube successfully. They found 96% success rate in pressure support of 13.2 CmH_2_O, but this study did not include neonates and patients less than 6 months and these groups were excluded from the study and it was recommended in this study that further studies are needed for various populations.

Olsen et al. (2002) completed a Cross over RCT on 14 neonates, in 4 hours courses including PSV + VG (volume Guarantee) and SIMV. In this study it was shown that minute ventilation was higher in PSV+VG than SIMV and mean pressure of airways was also higher in PSV+VG. No difference was observed in the dynamic compliance between the two methods, but the appropriate pressure level in PSV mode was not specified in this study.

Nayeri et al. (2009) randomly investigated 30 preterm infants during separation from mechanical ventilation with two PSV and SIMV mode and concluded that the duration of separating from the ventilator was significantly shorter in the PSV mode than SIMV, and no difference existed in extubation failure between these two modes. But, in this study, the used or proper pressure of PSV was undefined and our clinical trial is of the few studies that have included the proper pressure in PSV mode alone and the sample size is relatively more adequate.

Unlike Nayeri’s study that has reported no pneumothorax (Nayeri et al., 2009), in our study, the rate of pneumothorax during ventilation with PSV14 cmH_2_O was more, but no statistically significant difference could be observed. That might be because this pressure can cause pneumothorax when the patient has spontaneous inspiration and fewer pressures are safer in PSV mode.

In our study there was no significant difference between the tidal volumes of two groups. Indeed, in PSV mode, which varies the tidal volume, is the mechanic of respiration. Therefore the tidal volume could be more favorable by using volume assured PSV mode. Nafday et al (2005) conducted a survey on 25 premature infants and found that PIP was less in PSV+VG method than SIMV

The respiratory rate to tidal volume ratio was 2.8 in pressure of 10 CmH_2_O in our study which shows better patients’ condition for separating them from mechanical ventilator. However, no reliable number is available in neonatal population for this purpose (Nayeri, et al, 2009).

## 5. Limitations

Our study had also some limitations, We had low sample size for assessing the differences in secondary outcomes with lower incidence such as pneumothorax, and more researches of PSV with adequate sample sizes are warranted. Another limitation of our study was the limited follow-up time and not considering the consequences such as retinopathy of prematurity or intraventricular hemorrhage. The third limitation of this study was inability to use volume assured PSV mode.

## 6. Conclusion

The results of this study have shown that gradual decline of pressure and setting it to a less level of pressure in PSV mode can result in extubation success in infants and helps to prevent further complications of extubation failure. Though, further studies are needed to define the appropriate level of pressure in PSV mode in addition to acceptable tidal volume in neonates.
